# Comparison of Comprehensive Morphological and Radiomics Features of Subsolid Pulmonary Nodules to Distinguish Minimally Invasive Adenocarcinomas and Invasive Adenocarcinomas in CT Scan

**DOI:** 10.3389/fonc.2021.691112

**Published:** 2022-01-04

**Authors:** Lu Qiu, Xiuping Zhang, Haixia Mao, Xiangming Fang, Wei Ding, Lun Zhao, Hongwei Chen

**Affiliations:** ^1^ Department of Radiology, Wuxi People’s Hospital, Nanjing Medical University, Wuxi, China; ^2^ Department of Radiology, Wuxi Children’s Hospital, Nanjing Medical University, Wuxi, China; ^3^ Department of Intervention, Wuxi People’s Hospital, Nanjing Medical University, Wuxi, China; ^4^ Department of Research and Development, Deepwise Medical Artificial Intelligence Research Institute, Beijing, China

**Keywords:** radiomics, subsolid, MIAs, IACs, morphological

## Abstract

**Objective:**

To investigative the diagnostic performance of the morphological model, radiomics model, and combined model in differentiating invasive adenocarcinomas (IACs) from minimally invasive adenocarcinomas (MIAs).

**Methods:**

This study retrospectively involved 307 patients who underwent chest computed tomography (CT) examination and presented as subsolid pulmonary nodules whose pathological findings were MIAs or IACs from January 2010 to May 2018. These patients were randomly assigned to training and validation groups in a ratio of 4:1 for 10 times. Eighteen categories of morphological features of pulmonary nodules including internal and surrounding structure were labeled. The following radiomics features are extracted: first-order features, shape-based features, gray-level co-occurrence matrix (GLCM) features, gray-level size zone matrix (GLSZM) features, gray-level run length matrix (GLRLM) features, and gray-level dependence matrix (GLDM) features. The chi-square test and F1 test selected morphology features, and LASSO selected radiomics features. Logistic regression was used to establish models. Receiver operating characteristic (ROC) curves evaluated the effectiveness, and Delong analysis compared ROC statistic difference among three models.

**Results:**

In validation cohorts, areas under the curve (AUC) of the morphological model, radiomics model, and combined model of distinguishing MIAs from IACs were 0.88, 0.87, and 0.89; the sensitivity (SE) was 0.68, 0.81, and 0.83; and the specificity (SP) was 0.93, 0.79, and 0.87. There was no statistically significant difference in AUC between three models (p > 0.05).

**Conclusion:**

The morphological model, radiomics model, and combined model all have a high efficiency in the differentiation between MIAs and IACs and have potential to provide non-invasive assistant information for clinical decision-making.

## Introduction

Lung cancer ranks first in global cancer mortality (1). With the application of low-dose computed tomography (LDCT) in lung cancer screening, most of lung cancers can be detected at a very early stage. The proportion of adenocarcinoma mainly manifested as subsolid nodules has surpassed that of squamous cell carcinoma (2) and accounts for the highest incidence of malignant tumor of the lung. Subsolid nodules were found in about 0.2%–0.5% of people who underwent physical examination (3). Because of the limitations of radiology, the diagnosis of subsolid pulmonary nodules became a difficult problem. The World Health Organization (WHO) 2015 classified lung cancer as adenocarcinoma *in situ* (AIS), minimally invasive adenocarcinoma (MIA), and invasive adenocarcinoma (IAC).

Previous studies (4–6) mostly focused on distinguishing non-invasive (AISs) from invasive lesions (MIAs and IACs). However, as preinvasive lesions, AISs and MIAs have relatively good clinical prognosis with a 5-year survival rate close to 100% (7), which is 74.6% in IACs, showing intermediate and advanced clinical invasive behavior (8). Besides, clinical interventions are different. For MIAs, regular follow-up is recommended unless the diameter of pulmonary nodules is >1.5 cm or the patient is >70 years old (9). Segmentectomy or wedge-shaped resection can provide better recovery for MIAs (10). A multidisciplinary approach involving lobotomy and extensive lymphadenectomy is recommended for IACs. Correct diagnosis of MIAs and IACs is of great significance for the choice of surgery type and therapeutic schedule.

MIAs and IACs share a similar performance in CT scan. While AISs are often pure ground glass nodules (pGGNs), infiltrating lesions (MIAs and IACs) are both presented as mixed ground glass nodules (mGGNs). IACs may have more invasive CT findings than MIAs, but radiologists have certain bias in diagnosis because of subjective experience, and no quantitative evaluation criteria were published, so accurate diagnosis is very difficult.

Fast frozen pathology may help to select surgical methods due to its accurate “golden standard.” However, the anastomosis of pathology and paraffin sections was affected easily, and the low quality of fast sampling leads to magnification of heterogeneity of histopathological examination. For example, when adenoid tissue is found around scars, it is difficult to determine whether it is an invasive component or normal tissue (11). The International Association for The Study of Lung Cancer stresses that it is hard to make a definitive diagnosis without sampling all components of tissue.

As a process of high-throughput extraction of high-dimensional quantitative image features using automatic characterization data algorithms, radiomics creates associations between machine learning and medical images. It has made good progress in lung cancer, glioma, prostate cancer (12), and breast cancer in recent years. Some studies have used radiomics based on morphology to investigate feature analysis of lung adenocarcinoma and distinguish preinvasive lesions from invasive adenocarcinoma. However, the actual effect of these methods is still not clear, and the established model is difficult to explain due to the lack of connection with conventional sign science.

Our research aims to build a comprehensive morphological model, radiomics model, and combined model to investigate the diagnostic efficiency of lung infiltrate lesions presented as subsolid nodules. In addition, we compared and evaluated the performance among these three models.

## Materials and Methods

### Study Population

This retrospective study was approved by the Institutional Review Board with a waiver of informed consent. People who got CT chest scans and underwent complete surgical or underwent biopsy with confirmed MIA or IAC from January 1, 2010, to May 31, 2018, in our hospital were concluded in this study.

**Figure 1:**
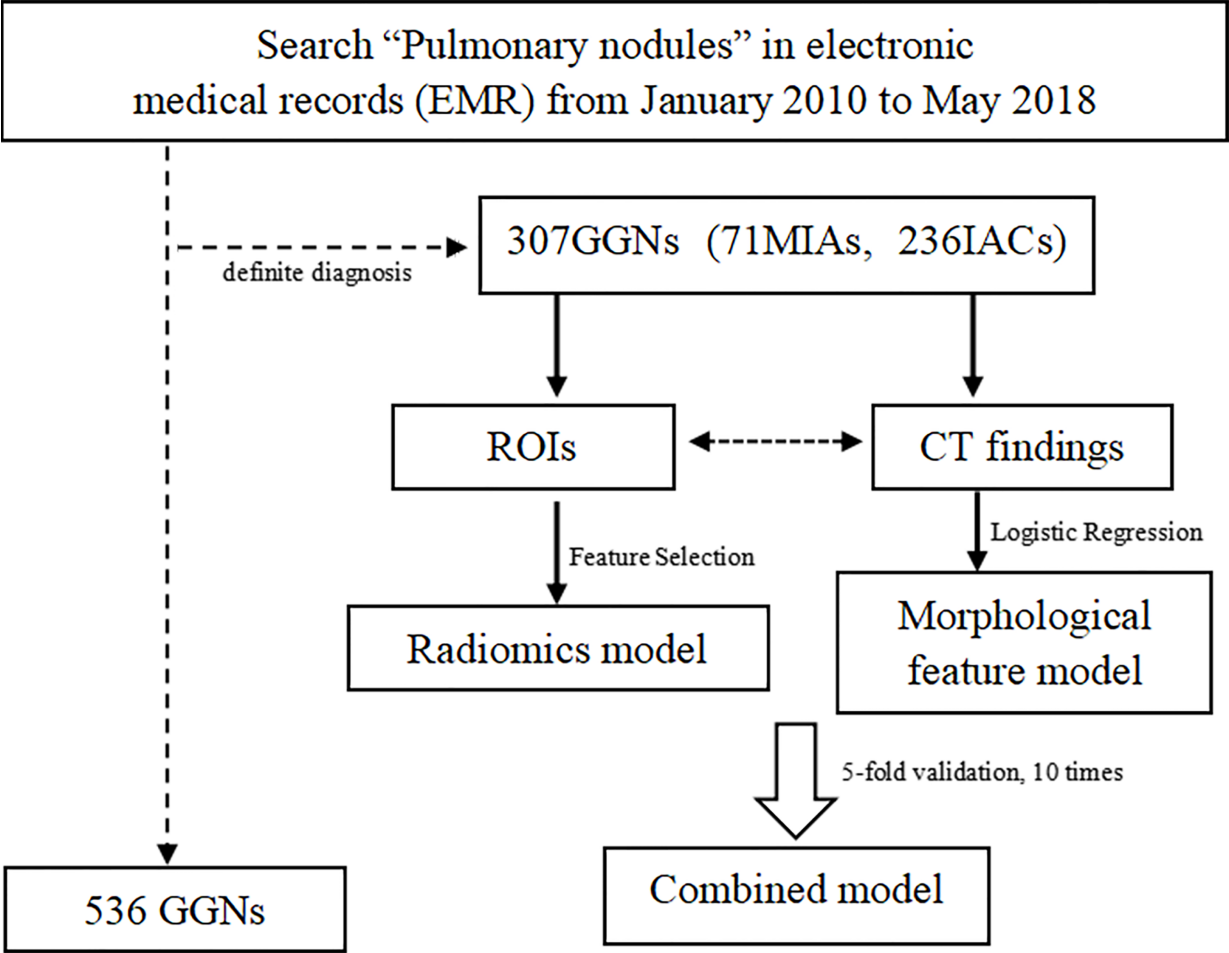
Flow diagram of the experiment.

The inclusion criteria are as follows: 1) subsolid nodules, maximum diameter ≤3 cm; 2) individuals with at least one thin-section CT study of thickness of 1 mm containing the GGNs; 3) the interval between the CT examination and pathology was less than 1 month. The exclusion criteria are as follows: 1) poor image quality affected observation; 2) puncture biopsy, radiofrequency ablation or systemic chemotherapy, or chest radiotherapy had been performed before CT examination; 3) the location of the lesion in pathological results was not clear. The arrangement process is shown in **Figure 1**. Finally, 307 GGNs constituted our study population.

### CT Examinations

Thin-section CT used for morphological and radiomics features analyses was performed using one of the following two scanners: the 128-layer Siemens Definition Flash Dual-source CT scanner and the 64-layer Siemens SOMATOM Definition Sensation Dual-source CT scanner (Germany) with 120 kVp, 180 mA, pitch 1.2:1, and FOV 30–34 cm. The conventional scanning layer has a thickness of 5 mm, layer spacing 5 mm, filter function C, and image matrix 512 × 512. Images were reconstructed using the Lung Sharp 70 reconstruction algorithm with a thickness of 1 mm, spacing 1 mm. Images were observed in pulmonary window (window width: 1,500 HU; window: -400 HU).

### Pathological Analysis

Before surgery, CT-guided puncture examination was performed to fix the anatomical location with the sampled corresponding materials, and the surgeon recorded the biopsy site after positioning. During operation, pathological lung tissue was removed based on the corresponding lung gross anatomy and CT images, and pathologist made a diagnosis and recorded the location.

All resected specimens were formalin fixed and stained with hematoxylin–eosin in accordance with the routine regulations of our hospital. Two pathologists with 3 and 10 years of experience in pathological diagnosis reviewed the specimens and recorded the pathological subtype according to the International Association for the Study of Lung Cancer (IASLC)/American Thoracic Society (ATS)/European Respiratory Society (ERS). When there was disagreement, consensus is reached through discussion. All GGNs of adenocarcinoma were divided into AIS, MIA, and IAC groups.

### Extraction of Quantitative Features of GGNs

Morphological features of GGNs were reviewed by two radiologists of 3 years of experience who were blinded to the histopathology diagnosis, which is more universal in practical terms. CT-based subjective findings were recorded in consensus. Following morphological features were detailed:

1. Lesion size: the average of major diameter and vertical short diameter measured on the largest section in the lung window (13);
2. Subtype (pGGN/mGGN);
3. Location (hilar/intrapulmonary/distance from pleura < 5 mm);
4. Shape (spherical/ellipsoid/irregular/crescent);
5. Margin (clear/fuzzy/partly clear): whether the edge can be clearly delineated with a thin line without magnification;
6. Lobulation (absence/shallow/deep);
7. Spiculation (absence/long/short);
8. Spinous sign (yes/no);
9. Solid component (uniform/diffuse small flake/diffuse large flake/circular or oval/others);
10. Cavity (yes/no);
11. Bronchogram (absence/normal/narrow/interrupted/distorted/expanded);
12. Vessel convergence (absence/vascular connections (normal)/vascular connections (enlargement)/vascular connections (cluster)/vascular coverage (normal)/vascular coverage (rigid)/vascular coverage (interrupt)/vascular coverage (enlargement)/vascular coverage (distortions)/vascular coverage (increase abnormal branch)/vascular compression/vascular adjacency;
13. Calcification (no/bulky/fine): >100 HU;
14. Fat ingredients (yes/no): -120 HU~-40 HU;
15. Liquid composition (yes/no): -30 HU~30 HU;
16. Satellite lesions (yes/no): small nodules < 5 mm around the main nodules;
17. Pleural indentation (no/adjacent (no traction or depression)/adjacent (thickened)/traction/depression);
18. Distal crescent emphysema (14) (yes/no).

**Figure 2:**
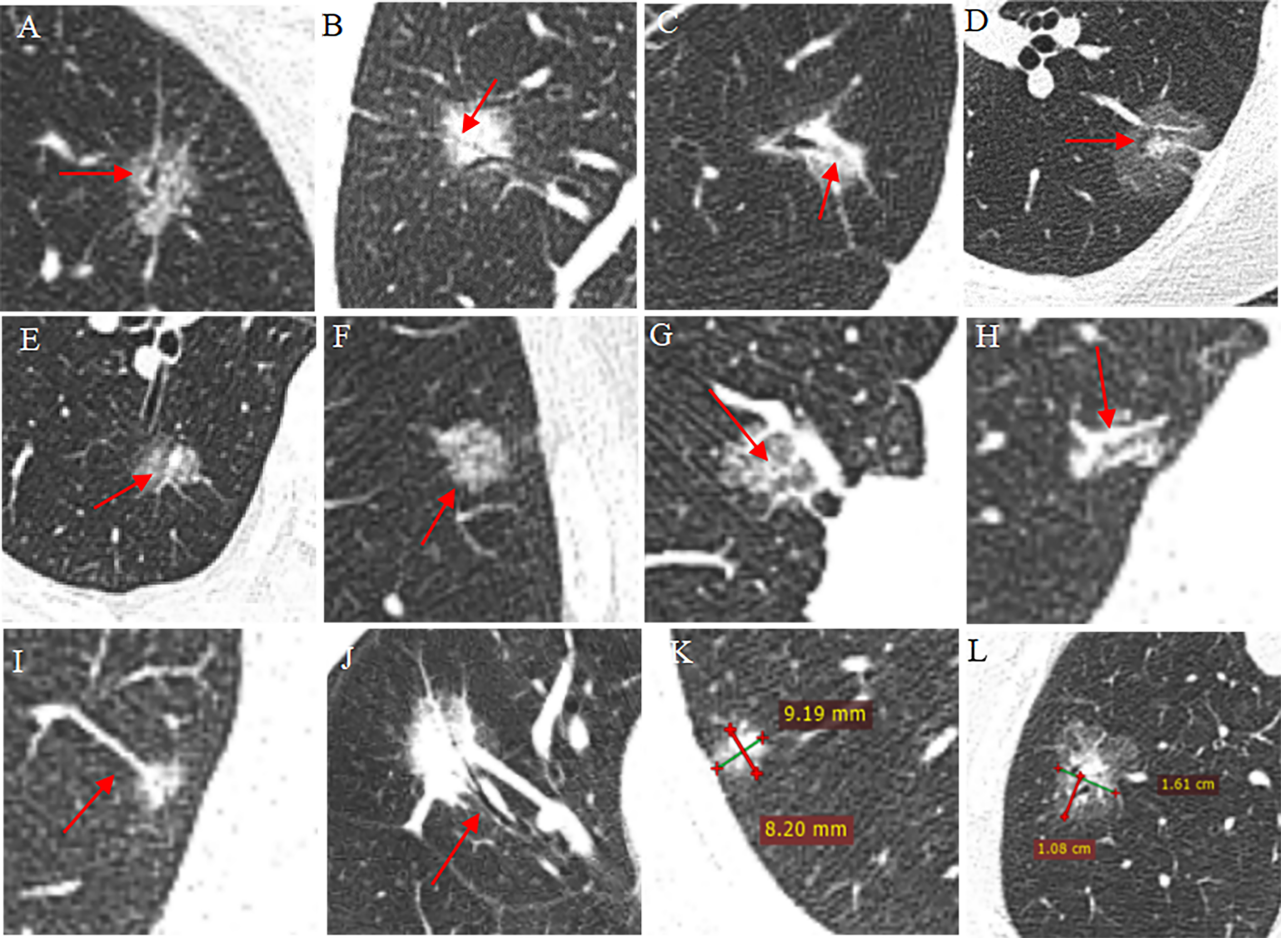
Part of the annotation schematic diagram of CT findings. **(A–C)** Bronchial sign: dilated **(A)**, stenosis **(B)**, interrupt **(C)**; **(D–F)** intra-nodule component: round **(D)**, big flake **(E)**, small patches **(F)**; **(G–J)** vessel sign: vessel cover sign_abnormal branches **(G)**, vessel cover sign_enlarged **(H)**, vessel connected_congestion **(I)**, convergence sign **(J)**; **(K, L)** quantitative measurement of size **(K)** and lobulation **(L)**.

A partial example is shown in **Figure 2**. In case of disagreement, the final decision will be made by a third radiologist with 10 years of experience in imaging diagnosis. The consistency of two CT readers was 0.76, p < 0.001.

Thin-section CT images covering GGNs were transferred and anonymized before being exported to the software. Nodule segmentation was performed using a semiautomatic software (Beijing Deepwise Company). Volumes of interest (VOIs) were drawn around the boundary of GGNs, operators were accurately adjusted to cover the entire lesion, then various radiomics features were calculated and extracted automatically. Analyzed radiomics features were as follows: (a) first-order features, (b) shape-based features, (c) GLCM features, (d) GLSZM features, (e) GLRLM features, and (f) GLDM features. The original VOI images were filtered by a wavelet operator or Gaussian operator to extract more features.

### Feature Selection and Signature Construction

To reduce data redundancy or selection bias in models, the following four methods were taken. First, The Pearson χ^2^ test was used to pick up significant morphological features. Second, the Pearson correlation coefficient r between any two features was calculated and when r >0.9, and the feature whose Pearson correlation coefficient with the dependent variable was lower was removed. Third, the least absolute shrinkage and selection operator (LASSO) was used to further select features by penalty parameter tuning and 10-fold cross-validation based on the minimum criteria. Finally, the “Select from model” method was used to keep helpful features, which is a meta-transformer that can go alongside any estimator that assigns importance to each feature through a specific attribute. The features considered unimportant if the corresponding importance of the feature values were below some provided threshold parameter were removed.

### Statistical Analysis

All statistical analyses were performed using SPSS ver. 25.0 (SPSS Inc., Chicago, IL, USA) and MedCalc ver. 12.0 (MedCalc Software, Mariakerke, Belgium).

The Kolmogorov–Smirnov test and Levene test were used to test the normality and homogeneity of data, respectively. Variables that satisfy this condition are expressed as mean ± standard deviation ( $\left(\bar{\chi }\pm \text{s}\right)$ ), while non-conforming variables are expressed as median. The independent sample t test was used when data satisfy normality and homogeneity; otherwise, the Mann–Whitney U test was used. The statistical differences of categorical variables were determined by the Pearson χ^2^ test, the continuity-corrected chi-square is tested when the theoretical number 1 ≤ T < 5 and sample size n ≥ 40, and Fisher’s exact method was used when T<1 or n<40. Characteristics with a p-value <0.05 were statistically significant.

AUC was used to evaluate diagnostic performance (including SP and SE of the model), but it is not comprehensive in clinic because it depends on whether to avoid false positive or negative benefits more. We use decision curve analysis (DCA) to find a net benefit decision. The Delong test analyzed and compared the AUC difference.

## Results

### Morphological Model in Discriminating MIAs and IACs

**Figure 3:**
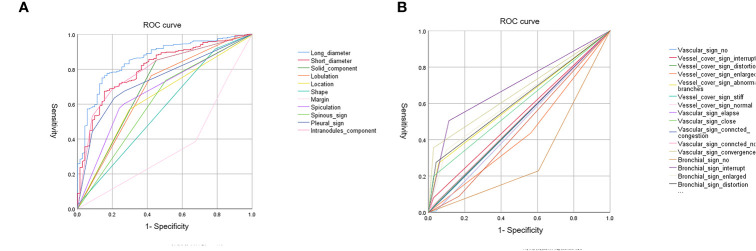
**(A, B)** ROC curves for CT findings.

A total of 307 subsolid nodules were retrospectively concluded in this study, including 71 MIAs and 236 IACs. Among comprehensive and systematic thin-section morphological features, lesion size, solid component, lobulation, location, shape, margin, spiculation, spinous sign, pleural sign, intra-nodule component, bronchial signs, and vascular signs were used as input variables for further logistic regression analysis (**Figure 3**). The comparison of these findings is shown in **Table 1**.

**Table 1:** Morphological findings in MIA and IAC groups.

Morphological features		MIA	IAC	p-value	Morphological features		MIA	IAC	p-value
Size (mm)	Long diameter			p = 0.00 *^a^*	Cavity	No	60	191	p = 0.49
	Short diameter			p = 0.00 *^a^*		Yes	11	45	
Solid component	pGGN	39	35	p = 0.00 *^a^*	Liquid component	No	71	236	p = 1
	mGGN	32	201			Yes	0	0	
Lobulation	No	9	7	p = 0.00 *^a^*	Satellite lesions	No	71	234	p = 1
	Shallow	32	49			Yes	0	2	
	Deep	30	180		pleura sign	No	52	77	p = 0.00 *^a^*
Calcification	No	71	236	p = 1		Close	1	0	
	Huge	0	0			Thicken	3	9	
	Small	0	0			traction	9	46	
Location	Intrapulmonary	50	101	p = 0.00 *^a^*		Indentation	6	104	
	<5 mm from the pleura	21	134		Spinous sign	No	35	62	p = 0.00 *^a^*
	Perihilar	0	1			Yes	36	174	
Shape	Sphere	4	5	p = 0.03 *^a^*	Spiculation	No	52	94	p = 0.00 *^a^*
	Oval	11	15			Short	17	136	
	Irregular	56	211			Long	2	6	
	Crescent	0	5		Intra-nodules component	Homogeneous	39	35	p = 0.00 *^a^*
Margin	Clear	10	59	p = 0.00 *^a^*		Round	4	10	
	Dizzy	48	91			Patches	8	36	
	Part-clear	13	86			Flake	14	30	
Vascular sign	No	3	2	p = 0.00 *^a^*		Others	6	125	
	Elapse	0	0		Bronchial sign	No	43	54	p = 0.00 *^a^*
	Close	0	0			Interrupt	8	119	
	Vessel convergence sign	2	84			Dilation	7	81	
Vascular sign_connected	Normal	0	3			Distortion	3	65	
	Congestion	26	86			Narrow	2	47	
Vascular sign_vessel cover sign	Interrupt	2	19			Normal	12	21	
	Enlarged	40	103		Distal crescent emphysema	No	69	215	p = 0.12
	Distortion	9	29			Yes	2	21	
	Normal	16	17		Fat component	No	71	236	p = 1
	Branches	4	64			Yes	0	0	
	Stiff	4	15						

a Indicates the difference was statistically significant.

After binary logistic regression analysis, thin-section morphological features of long diameter, short diameter, vessel_cover_sign_abnormal_branches, vascular_sign_convergence, solid component, and margin proved to be significant in differential diagnosis.

**Figure 4:**
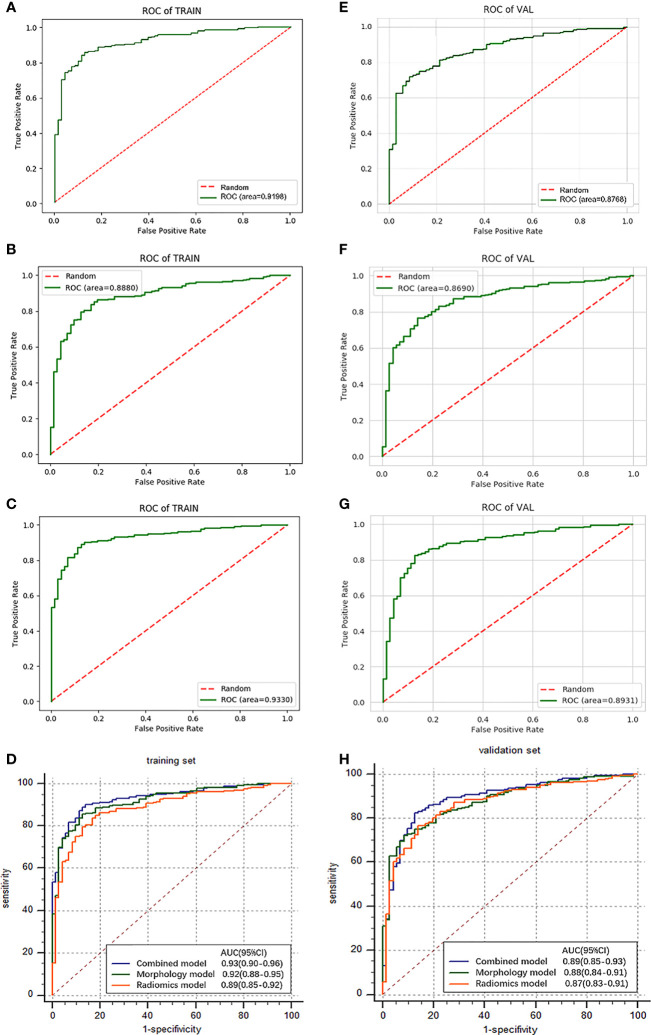
Results and comparison **(D, H)** of ROC curve analysis. The ROC curves for morphological model **(A)**, radiomics model **(B)**, and combined model **(C)** in the training cohorts. The ROC curves for morphological model **(E)**, radiomics model **(F)**, and combined model **(G)** in the validation cohorts.

The ROC curve evaluates the diagnostic performance of the morphological model, with AUC of training cohorts as 0.92 (sensitivity: 0.68, specificity: 0.97) (**Figure 4A**) and validation cohorts as 0.88 (sensitivity: 0.68, specificity: 0.93) (**Figure 4E**).

### Radiomics Feature Selection and Radiomics Signature Building

The algorithm extracted 1,259 features from all the VOIs, including 41 basic image data features and 1,218 radiomics features. There were 938 features with linear dependent coefficient>0.9, so 280 features were retained for Logistic Regression modeling. The model was initialized with parameters: “penalty”: “L1”, “C”: 1, “Fit_intercept”: True, “class_weight”: “Balanced”, “solver”: “liblinear”, “multi_class”: “auto”, C=0.01. Three most important texture features were selected, and their weights are shown in **Table 2**. We provide the coefficient of each selected feature. In logistic regression of the machine learning algorithm, we can convert the odds ratio (OR) value to the coefficient in the following ways:


$${\text{OR}}_{\text{Browse}}=\exp(\text{coef})$$

**Table 2:** The selected features in radiomics model and weight index.

Feature	coef	relative_to_max
wavelet-HHH_glcm_Imc2	-1.08	-1.00
wavelet-LHH_glcm_Imc2	-0.52	-0.48
wavelet-HLH_gldm_LowGrayLevelEmphasis	-0.48	-0.44

The coefficient values shown in the table are based on the features of the model built on 100% of the data. Five-fold cross validation was used to make sure 80% cases randomly formed training cohorts and 20% formed validation cohorts. Repeat the process for 10 times to minimize sampling error.

The results show a significant difference between MIAs and IACs. The AUCs of the training cohorts (**Figure 4B**) and validation cohorts (**Figure 4F**) were 0.89 (specificity: 0.83, sensitivity: 0.83) and 0.87 (specificity: 0.79, sensitivity: 0.81).

### Construction and Validation of Combined Model

To improve the classification performance, we fuse morphological features and radiomics features and select stable and effective ones by the LASSO method. The established features of high stability and identification efficiency were incorporated and presented as a combined feature set. Adjust the parameters to build an efficient individualized prediction model. We follow the principle of “result optimization.” When constructed models have similar AUC values, the one with fewer features is preferentially selected, because we try to use fewer features to achieve a higher diagnostic efficiency. As can be seen, 9 morphological features and 4 radiomics features were covered into a combined model due to the complicated interactive effect between features (**Table 3**). The obtained combined model training cohorts had an AUC value of 0.93 (sensitivity: 0.84, specificity: 0.90) (**Figure 4C**), and the validation cohorts had an AUC value of 0.89 (sensitivity: 0.83, specificity: 0.88) (**Figure 4G**).

**Table 3:** The selected features in combined model and weight index.

	Feature	coef	relative_to_max
1	Short diameter	1.64	-1
3	wavelet-HHH_glcm_Imc2	1.47	-0.90
14	Vessel convergence sign	1.02	0.62
7	wavelet-HHH_glcm_Imc1	0.99	-0.60
0	Long diameter	0.99	0.60
12	Vessel cover sign (interrupt)	0.61	-0.37
6	wavelet-HLH_gldm_LowGrayLevelEmphasis	0.48	-0.29
5	wavelet-LHH_glcm_Imc2	0.47	-0.29
8	Spiculation (short)	0.43	0.26
13	Vessel cover sign (abnormal branches)	0.43	0.26
10	Bronchial sign(dilation)	0.31	0.19
9	Bronchial sign(no)	0.15	-0.09
11	Bronchial sign(normal)	0.08	-0.05

### Comparison of Simple Signatures and Combined Signature

In training cohorts, the morphological model achieved an AUC value of 0.92 (sensitivity: 0.68, specificity: 0.97), the radiomics model showed a lower AUC of 0.89 (sensitivity: 0.83, specificity: 0.83), and the combined model got the highest AUC as 0.93 (**Figure 4D**). In validation cohorts, the morphological model achieved an AUC value of 0.88 (sensitivity: 0.68, specificity: 0.93), the radiomics model showed a lower AUC of 0.87 (sensitivity: 0.81, specificity: 0.79), and the combined model got the highest AUC as 0.89 (**Table 4** and **Figure 4H**). Introducing morphological findings into the radiomics model improved the performance. However, the Delong analysis showed no significant difference in pairs with p of 0.06, 0.08, and 0.46, greater than 0.05.

**Table 4:** Comparison of morphological model, radiomics model, and combined model.

	Morphological model (Model 1)	Radiomics model (Model 2)	Combined model (Model 3)
Feature selection	Chi-square + F1 test	LASSO	LASSO
Algorithm	LR	LR	LR
AUC	0.88	0.87	0.89
Delong analysis	Model 1 VS. Model 2	Model 2 VS. Model 3	Model 3 VS. Model 1
	p = 0.06	p = 0.08	p = 0.46

**Figure 5:**
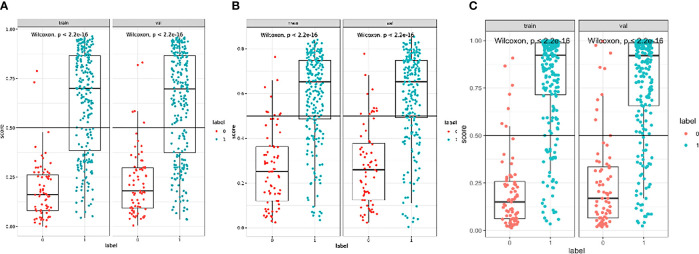
The boxplots of morphological model **(A)**, radiomics model **(B)**, and combined model **(C)**.

**Figure 6:**
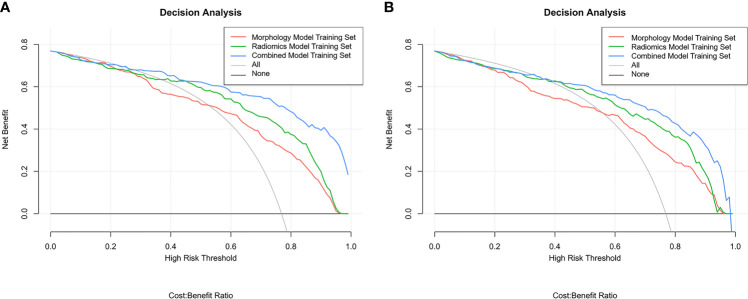
The DCA comparison of training cohorts **(A)** and validation cohorts **(B)**.

The boxplots (**Figure 5**) revealed the dispersion of samples which indicated the sample score in combined model was more concentrated and had better efficiency than the other two models. Within the approximate range of the threshold of 0.1–1.0, the curves of three models are all above the gray line (two extreme cases: all samples were interfered or not interfered). They all have good decision-making efficiency for the differentiation of MIAs and IACs. The radiomics model is better than the morphological model, and the combined model achieved the best decision efficiency (**Figure 6**).

## Discussion

In the present study, three individual models were constructed to distinguish MIAs and IACs in patients with subsolid pulmonary nodules preoperatively. By extracting high-throughput quantitative descriptors from routinely acquired CT images, the radiomics model provides a non-invasive tool to improve routine lung cancer diagnosis. Besides, we established a comprehensive CT imaging system including 18 types of morphological features and 63 subtypes to accurately characterize the morphology of GGNs. The basic characteristics, internal composition, and growth pattern of pulmonary nodules are well documented, simulating the real factors imaging physicians would consider in the process of diagnosis. Morphological features and radiomics features decode phenotypes of GGNs differently; we integrated these quantitative data to construct a combined model to improve the performance and speed up the implementation of personalized medicine.

Previous literature (15) showed that the AUC of the radiomics model was 0.85, higher than that of the morphological model (AUC: 0.79), lower than that of the combined model (AUC: 0.89) due to the not comprehensive evaluation for lesions. In our study, the AUC of the morphological model was a little higher than that of the radiomics model, although the difference was also not statistically significant. Some experts believe that a set of clinically relevant signs easily got a high score in determining the risk of pulmonary nodules effectively, and the morphological model determined by experienced radiologists is likely to be more effective than the radiomics model (16). Besides, our study included a range of morphological findings, including some rare ones, to assess the growth of GGNs comprehensively, thus playing a better role in predicting properties compared with few features.

A previous study (15) revealed two first-order histogram features; one GLSZM-based feature, one morphological feature, and one roughness feature were predictors for distinguish MIAs from IACs. In our study, short diameter and vessel convergence sign got the first and third weights, respectively, partly consistent with previous studies (17), which shows that the diameter significantly increased in the GGN follow-up. Besides, three GLCM-based features and one GLDM-based feature (low gray-level emphasis) got a high weight and were selected for the combined model. GLCM statistically describes texture where two pixels keeping a certain distance on the image have a certain gray level, namely, the spatial correlation characteristics of the gray level. Prior literatures have found that GLCM-based features were important in the categorization (18) and recognition of lung tumor, even superior to histogram (19). Wu (20) believed that the GLCM-based feature (especially GLCM_Entropy_log10) was the predictor for histological invasiveness of the pulmonary adenocarcinoma spectrum, partly consistent with our study that GLCM-based features play an important role and more detailed research is needed.

Besides, our study proved that GLDM_ low gray level emphasis was an important indicator for distinguishing MIAs from IACs in subsolid nodules; studies also proved that low gray level emphasis helps to differentiate lung adenocarcinoma from another lung cancer histological type (21) and predict NSCLC survival (22).

When we did five-fold cross validation, we trained the model five times on 80% of the data and tested on 20%. We ensure that each data point ends up in the 20% test set. We have therefore used every data point we have to contribute to an understanding of how well our model performs the task of learning and predicting. The purpose of cross-validation is model checking, so 100% of the data was used to build a new separate model whose parameters are the same with five-fold cross validation models. Moreover, the reported performance results came from the fusion of cross-validation models. The model’s performance can be estimated according to the Central Limit Theorem. We believe that it is a good estimator of the final model’s performance on future unseen data.

Several limitations in this study should be addressed in the future. As a retrospective study that selected only lesions in patients who underwent surgery or biopsy, the performance of the proposed model may be overestimated. Prospective or external validation studies were warranted. Second, all sub-solid pulmonary nodules ≤3 cm were included in this study, but pulmonary nodules <4 mm are considered unimportant in clinical practice, and small volume may affect the extraction and calculation of radiomics features. Third, CT examinations were all performed in our hospital; multicenter validation is needed for generalization of the model.

## Conclusion

1. A comprehensive systematic morphological model shows good predictive performance in MIAs and IACs, which is better than the radiomics model;
2. The radiomics model distinguishes MIAs and IACs effectively, but its diagnostic efficiency is lower than those of the morphological model and combined model, especially GLCM and GLDM features;
3. The combined model of integrated features shows no better performance in diagnosing MIAs and IACs, but the combined model achieved the best decision efficiency.

In conclusion, the morphological model, radiomics model, and combined model all have high diagnostic efficacy in the differentiation of MIAs and IACs, although the difference was not statistically significant.

## Data Availability Statement

The raw data supporting the conclusions of this article will be made available by the authors, without undue reservation.

## Ethics Statement

Ethical review and approval were not required for the study on human participants in accordance with the local legislation and institutional requirements. Written informed consent for participation was not required for this study in accordance with the national legislation and the institutional requirements.

## Author Contributions

XF contributed to the conception and design of this study. LQ and HM contributed to the data acquisition and manuscript editing. XZ and WD contributed to the data interpretation and analysis. HC contributed to the study supervision. LZ contributed to radiomics method guidance. All authors contributed to the manuscript review. All authors contributed to the article and approved the submitted version.

## Funding

This work was supported by the research rants from the Jiangsu Province Natural Science Foundation (No. BK20191143, X.M. Fang), National Natural Science Foundation of China (No. 81271629, XF), 333 High-level Talents Training Key Project of Jiangsu (No. BRA2018013, XF), Wuxi Medical innovation Team Program(No. CXTD002, XF), and the Medical Expert Team Program of Wuxi Taihu Talent Plan 2021.

## Conflict of Interest

The authors declare that the research was conducted in the absence of any commercial or financial relationships that could be construed as a potential conflict of interest.

## Publisher’s Note

All claims expressed in this article are solely those of the authors and do not necessarily represent those of their affiliated organizations, or those of the publisher, the editors and the reviewers. Any product that may be evaluated in this article, or claim that may be made by its manufacturer, is not guaranteed or endorsed by the publisher.
